# Cancer increases the risk of atrial fibrillation during long-term follow-up (OPERA study)

**DOI:** 10.1371/journal.pone.0205454

**Published:** 2018-10-05

**Authors:** Helena Kattelus, Y. Antero Kesäniemi, Heikki Huikuri, Olavi Ukkola

**Affiliations:** Research Unit of Internal Medicine, Medical Research Center Oulu, Oulu University Hospital, and University of Oulu, Oulu, Finland; University of Tampere, FINLAND

## Abstract

**Introduction:**

Relation between atrial fibrillation (AF) and cancer is known but not very well understood. The purpose of this prospective study was to find out whether subjects with cancer were at greater risk of AF than subjects without cancer.

**Materials and methods:**

The study was based on the OPERA (Oulu Project Elucidating Risk of Atherosclerosis) material and had 1045 subjects and the mean follow-up time of 16.3 years. During the follow-up AF and cancer diagnosis were made (atrial flutter included) if these events were listed in the National Death Registry and/or hospital discharge registry.

**Results:**

In this study 130 subjects (12%) had cancer and 19% of these had AF, whereas only 9% of those without cancer experienced AF during the follow-up (p<0.001). Subjects in the cancer group had greater probability of developing atrial fibrillation during the follow-up time in comparison to the subjects without cancer (Hazard ratio (HR) 2.47 (95%CI) 1.57–3.88) in multivariate model including relevant confounding factors.

**Conclusion:**

The main finding of this OPERA study was that cancer is an independent risk factor of atrial fibrillation. Still it remains unclear whether this association is causative or whether cancer and atrial fibrillation just share the same pathophysiologic mechanisms.

## Introduction

Atrial fibrillation (AF) is the most common sustained cardiac arrhythmia. It affects 1.5% to 2% of the general population and its prevalence and incidence are both expected to rise in the future [1][2]. Patients with AF have increased mortality and morbidity and impaired quality of life. They also have many cardiovascular risk factors, structural heart disease and comorbidities. [3] Age, hypertension, heart failure, coronary artery disease (CAD), valvular heart disease, obesity, diabetes mellitus and chronic kidney disease are all conditions that increase the prevalence of AF [4]. Even in the absence of pre-existing cardiovascular disease or valvular conditions, AF doubles the mortality risk in men and women [5].

It is known that cancer patients suffer from different kinds of comorbidities [6]. It has recently been found that cancer has an association with atrial fibrillation. In recent years, few large cohort studies have strengthened the epidemiological evidence of AF in cancer patients [7]. However, most of the previous studies have focused on postoperative AF, especially AF after lung cancer surgery [8][9]. In addition, there are also studies of AF in colorectal and breast cancer [10], and similar associations have reported from Denmark with cancers of the lung, kidney, colon, ovary, and on non-Hodgkin´s lymphoma [11]. It remains unclear in which mechanism AF is linked to cancer. Potential pathogenetic mechanisms that have been suggested are direct effect of the tumor, medical therapy, surgery, inflammation, paraneoplastic manifestation, imbalance in autonomous nervous system, hypoxia, metabolic and electrolyte disorders, aging, and other cancer-related comorbidities. [1][7]

The purpose of this prospective study was to find out whether subjects with cancer were at greater risk of AF than subjects without cancer. The study was based on the OPERA (Oulu Project Elucidating Risk of Atherosclerosis) material and had 1045 subjects.

## Materials and methods

### Study population

In the OPERA study, middle-aged hypertensive subjects and age- and sex-matched control subjects were randomly selected from the national registries in the early 1990s [12]. The total number of subjects was 1,045. The study was designed to evaluate the risk factors and the occurrence of atherosclerotic cardiovascular diseases. All study subjects visited the research laboratory of the Department of Internal Medicine, University of Oulu, where they went through a clinical examination and a wide range of routine laboratory analyses were taken. At this first visit, subjects filled in a standardized health questionnaire covering the past medical history, alcohol consumption, smoking, physical activity, current and former medication and family history. The questionnaire was completed by two specially trained nurses and the details were checked by the physician later during the same visit. The study was approved by the Ethics Committee of the Medical Department of the University of Oulu.

### Follow-up

During the follow-up [13] AF or cancer diagnosis were made (atrial flutter included) if these events were listed in the National Death Registry and/or hospital discharge registry (HILMO). Diagnosis of AF based on standard 12-lead resting ECG. The follow-up time lasted until December 31, 2009 or to the occurrence of the first event. The mean follow-up time was 16.3 years (median 17.6 years, range 0–19 years).

### Laboratory tests and echocardiographic methods

All the laboratory test samples were obtained after an overnight fast [14]. A Hewlett-Packard ultrasound color system, Sonos 500 (Hewlett-Packard Company, Massachusetts, USA) was used for the echocardiographic examinations. All procedures were performed by one experienced cardiologist (Markku Ikäheimo), who was blinded to the other data and grouping of the study subjects. M-mode measurements were obtained under 2-D guidance according to the recommendations of the American Society of Echocardiography [15].

### Statistical methods

The statistical significances of differences in continuous and categorical variables between the subjects with and without cancer and AF were assessed using the standard t-test and the chi-square test, respectively. Logarithmic transformations were used when variable distributions were not normal (atrial natriuretic peptide (ANP), high-sensitive C-reactive protein (hs-CRP), triglycerides, alanine aminotransferase (ALT), creatinine). The cumulative proportional probability of the development of AF requiring hospitalization is shown by the Kaplan-Meier curves. The Log Rank test was used to evaluate the statistical significance of the separation of the curves. The Cox hazards model was used to evaluate the univariate and multivariate significance of different factors in predicting new-onset AF requiring hospitalization. The data were analyzed using the IBM Statistics software 22. A p-value <0.05 was considered to be statistically significant.

## Results

The baseline features of patients with and without cancer (n = 1045) are shown in Table 1 and the same data for those with and without atrial fibrillation in Table 2. There were 130 subjects (12%) with cancer events during follow-up and 105 subjects (10%) with atrial fibrillation. The occurrence of atrial fibrillation events among cancer and non-cancer subjects was 19% and 9%, respectively (p<0.001).

**Table 1:** Baseline characteristics of the study subjects (n = 1045) according to the presence or absence of cancer.

	No cancer (n = 915)	Cancer (n = 130)	p-value
Age (years)	51 ± 6	53 ± 5	0.008
Sex (male), n (%)	444 (48%)	76 (58%)	0.034
Atrial fibrillation, n (%)	80 (9%)	25 (19%)	<0.001
Study group (hypertensives), n (%)	452 (49%)	67 (52%)	0.648
Diabetics, n (%)	98 (11%)	8 (6%)	0.107
Coronary artery disease, n (%)	74 (8%)	12 (9%)	0.718
Systolic blood pressure (mmHg)	148 ± 22	150 ± 22	0.344
Diastolic blood pressure (mmHg)	89 ± 12	90 ± 12	0.254
Body mass index (kg/m^2^)	27.7 ± 5	27.7 ± 4	0.911
Waist circumference (cm)	90 ± 13	92 ± 13	0.307
Fasting glucose (mmol/l)	4.8 ± 1.5	4.6 ± 1.0	0.239
ANP (pmol/l)	281 ± 169	283 ± 157	0.899
Total cholesterol (mmol)	5.7 ± 1.0	5.8 ± 1.1	0.439
HDL-cholesterol (mmol/l)	1.4 ± 0.4	1.3 ± 0.4	0.363
LDL-cholesterol (mmol/l)	3.5 ± 0.9	3.5 ± 1.0	0.803
Triglycerides (mmol/l)	1.6 ± 1.0	1.7 ± 1.3	0.242
hs-CRP (mg/l)	3.6 ± 6.8	4.9 ± 11.1	0.074
ALT (U/l)	32 ± 24	31 ± 16	0.653
Creatinine (μmol/l)	82 ± 33	83 ± 15	0.734
Alcohol (g/week)	60 ± 90	67 ± 88	0.383
Smoking (pack years)	9 ± 14	13 ± 15	0.003
IMT (mm)	0.87 ± 0.18	0.91 ± 0.22	0.033
Left atrial diameter (mm)	39 ± 5	40 ± 5	0.021
β-blockers, n (%)	250 (27%)	36 (28%)	0.929
Calcium blockers, n (%)	109 (12%)	19 (15%)	0.379
ACE-inhibitors, n (%)	174 (19%)	30 (23%)	0.274
Diuretic drugs, n (%)	144 (16%) 4%	28 (22%)	0.095
Digitalis, n (%)	19 (2%)	5 (4%)	0.208
Lipid lowering drugs, n (%)	27 (3%)	3 (2%)	0.681
Aspirin, n (%)	51 (6%)	7 (5%)	0.930

The values are means ± SD, absolute numbers with percentages or percentages alone. The medication data is expressed as number of subjects and percentages. Differences were tested by the ANOVA test for continuous variables and Pearson Chi-Squared test for categorical variables. ANP, atrial natriuretic peptide; hs-CRP, high-sensitive C-reactive protein; ALT, alanine aminotransferase; IMT, intima-media thickness; ACE, angiotensin converting enzyme.

**Table 2:** Baseline characteristics in the subjects (n = 1045) with and without atrial fibrillation (AF) in the follow-up.

	No AF (n = 940)	AF (n = 105)	p-value
Age (years)	51 ± 6	54 ± 5	<0.001
Sex (male), n (%)	455 (48%)	65 (62%)	0.009
Study group (hypertensives), n (%)	460 (49%)	59 (56%)	0.159
Diabetics, n (%)	92 (10%)	14 (13%)	0.254
Coronary artery disease, n (%)	92 (10%)	26 (25%)	<0.001
Systolic blood pressure (mmHg)	147 ± 22	156 ± 23	<0.001
Diastolic blood pressure (mmHg)	89 ±12	91 ± 14	0.138
Body mass index (kg/m^2^)	27.6 ± 5	29.0 ± 5	0.003
Waist circumference (cm)	90 ± 13	95 ± 13	<0.001
Fasting glucose (mmol/l)	4.7 ± 1.5	4.9 ± 1.3	0.434
ANP (pmol/l)	273 ± 153	350 ± 256	<0.001
Total cholesterol (mmol/l)	5.7 ± 1.1	5.7 ± 1.0	0.619
HDL-cholesterol (mmol/l)	1.4 ± 0.4	1.3 ± 0.4	0.021
LDL-cholesterol (mmol/l)	3.6 ± 1.0	3.6 ± 0.9	0.477
Triglycerides (mmol/l)	1.6 ± 1.0	1.8 ± 1.1	0.064
hs-CRP (mg/l)	3.8 ± 7.6	3.9 ± 6.0	0.868
ALT (U/l)	31 ± 23	37 ± 22	0.012
Creatinine (μmol/l)	82 ± 15	91 ± 89	0.007
Alcohol (g/week)	59 ± 86	79 ± 117	0.033
Smoking (pack years)	10 ± 14	13 ± 17	0.020
IMT (mm)	0.87 ± 0.18	0.92 ± 0.21	0.007
Left atrial diameter (mm)	39 ± 5	41 ± 5	<0.001
β-blockers, n (%)	247 (26%)	39 (37%)	0.018
Calcium blockers, n (%)	113 (12%)	15 (14%)	0.502
ACE-inhibitors, n (%)	174 (19%)	30 (29%)	0.014
Diuretic drugs, n (%)	153 (16%)	19 (18%)	0.634
Digitalis, n (%)	12 (1%)	12 (11%)	<0.001
Lipid lowering drug, n (%)	27 (3%)	3 (3%)	0.993
Aspirin, n (%)	47 (5%)	11 (10%)	0.020

The values are means ± SD, absolute numbers with percentages or percentages alone. The medication data is expressed as number of subjects and percentages. Differences were tested by the ANOVA test for continuous variables and Pearson Chi-Squared test for categorical variables. ANP, atrial natriuretic peptide; hs-CRP, high-sensitive C-reactive protein; ALT, alanine aminotransferase; IMT, intima-media thickness; ACE, angiotensin converting enzyme.

In the cancer group the proportion of males was 58% and in the non-cancer group 48% (p = 0.034). The mean age in patients in the cancer group was 53 years whereas in the non-cancer group it was 51 years (p = 0.008). The patients with cancer smoked more (p = 0.003). As regards to echocardiographic measurements, cancer patients had a greater left atrial diameter (p = 0.021) and carotid intima-media thickness (p = 0.033) than those without cancer.

Several baseline characteristics differed in the subjects with atrial fibrillation in the follow-up compared to those without AF. This is shown in Table 2. In the AF group the proportion of males was 62% and in the non-AF group 48% (p = 0.009). Also, the subjects with AF were older (p<0.001), more obese (p = 0.003), had more abdominal fat (p<0.001) and higher systolic blood pressure (p<0.001). The subjects in AF group had also higher ALT (p = 0.012), creatinine (p = 0.007) and ANP (p<0.001) values. In addition, HDL-cholesterol level (p = 0.021) was lower and CAD (p<0.001) was more prevalent in the AF group compared to the non-AF group. The subjects with AF also smoked more (p = 0.020) and used more alcohol (p = 0.033) than those without AF. The use of different types of medicines was analyzed and statistically significant differences between the AF and non-AF group were observed in the use of beta-blockers (p = 0.018), ACE-inhibitors (p = 0.014), digitalis (p<0.001) and ASA (p = 0.020). Furthermore, subjects belonging to the AF group showed larger left atrial diameter (p<0.001) and thicker intima-media layer (p = 0.007) compared to those without atrial fibrillation. There was no statistical difference in the distribution of the original OPERA study group (hypertension or control group) or diabetes status in AF and non-AF groups.

In multivariate Cox regression analysis of the predictors of atrial fibrillation, see Table 3, relevant covariates were chosen as potential confounding factors on the basis of their significance in univariate analyses. In addition to cancer event, the following additional factors were included in the model: age, sex, IMT thickness, systolic blood pressure, BMI, waist circumference, ANP, HDL-cholesterol, serum ALT concentration, serum creatinine, alcohol consumption, smoking (according to pack-years), left atrial diameter, use of beta-blocker, ACE-inhibitor, digitalis or aspirin. In this model, cancer event (p<0.001), age (p<0.001), ANP (p<0.001) and use of ACE-inhibitor (p = 0.003) or digitalis (p<0.001) were independently associated with AF.

**Table 3:** Baseline characteristics that in the multivariate analysis significantly predicted new-onset atrial fibrillation requiring hospitalization during follow-up.

	HR	95% CI	p-value
Age	1.07	1.03–1.11	<0.001
ANP	1.00	1.001–1.002	<0.001
ACE-inhibitors	0.52	0.34–0.81	0.003
Digitalis	0.18	0.10–0.34	<0.001
Cancer	2.47	1.57–3.88	<0.001

CI = confidence intervals, HR = Hazards ratios obtained from the Cox regression. The abbreviations are the same as in the Table 2. Baseline characteristics in the Table 2 that differed at the significance level p<0.05 between subjects with and without atrial fibrillation requiring hospitalization during follow-up were tested in the Cox hazards model.

**Fig 1:**
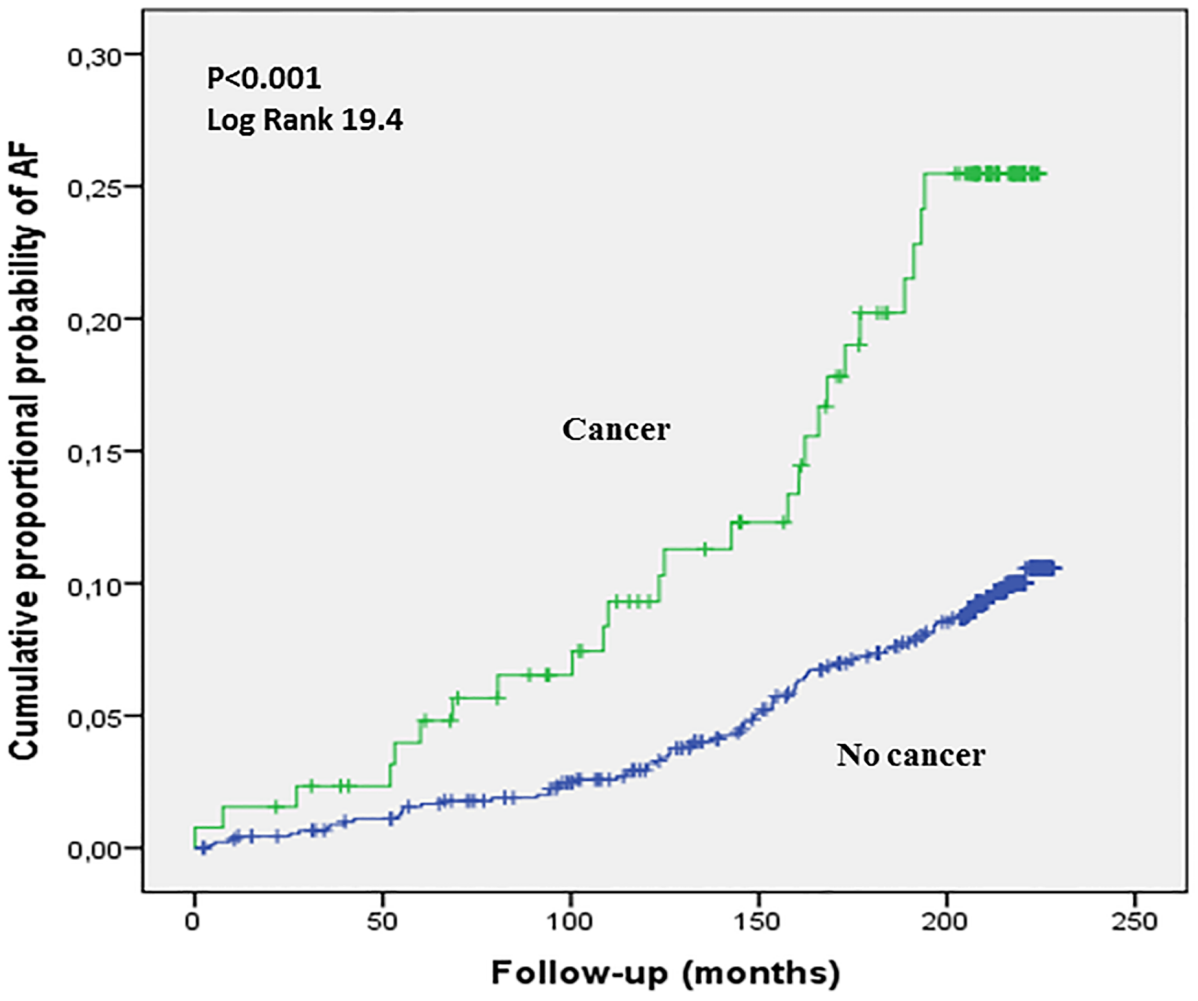
The cumulative proportional probability of AF in the cancer group.

Subjects in the cancer group had greater probability of developing atrial fibrillation during the follow-up time in comparison to the subjects without cancer (Hazard ratio (HR) 2.47 (95%CI) 1.57–3.88). After the follow-up time, 19.2% of subjects (n = 25) with cancer at the baseline had been diagnosed with atrial fibrillation, while 8.7% of subjects (n = 80) without cancer had AF. The difference between cancer and non-cancer group was statistically significant (p<0.001). The detailed cumulative proportional probability of AF in the cancer group is shown in Fig 1.

## Discussion

The main finding of this OPERA study, including over 1,000 subjects and a long follow-up time, was that cancer is an independent risk factor of atrial fibrillation. Still it remains unclear whether this association is causative or whether cancer and atrial fibrillation just share the same pathophysiologic mechanisms. Previous studies have suggested that cancer itself is a comorbid condition predisposing to AF.

The evidence of the association between cancer and atrial fibrillation is still slightly limited, apart from several studies of AF after lung cancer surgery. A large epidemiological study with 24,125 subjects was the first to show that AF is related to poor prognosis in cancer patients [16]. Some of the studies have focused on patients with colorectal and breast cancer. In a cohort study of 2,339 patients the prevalence of AF was two-folded among colorectal and breast cancer patients compared to controls who admitted for non-neoplastic surgery [10]. Another large population-based case-control study suggested that colorectal cancer patients, as well as patients diagnosed with some other cancer, have an increased risk for AF exclusively in the first 90 days after cancer diagnosis [17]. REGARDS-study instead concentrated on patients with a history of non-life-threatening cancer and those who did not require active cancer treatment. Actively treated cancer patients were excluded from the study. However, AF was still more prevalent in the cancer group (adjusted OR 1.19, 95% confidence interval 1.02–1.38). [9]

How could cancer increase the risk for atrial fibrillation? As seen in our study, both entities shared common risk factors, like age, sex and smoking. In addition, left atrial diameter associated with FA, and IMT thickness reflecting early atherosclerosis, were increased in both conditions. However, although these factors were adjusted for, the association between AF and cancer remained. Therefore, additional mechanism must be involved. In earlier studies, cancer surgery, medical therapy, inflammation, autonomic nervous system imbalance, paraneoplastic manifestations and other cancer-related comorbidities are among the mechanisms reported to be associated with increased atrial fibrillation risk among cancer patients [1][5][7].

As said before, the most studied type of AF is the postoperative AF (POAF), especially after pulmonary resection for lung cancer [18][19]. Even 12.6% of 13,906 patients who underwent lung cancer surgery developed POAF [20]. Also several cytotoxic drugs used for the treatment of cancer have been found to induce AF [21]. As a limitation, we do not have any information on the treatment modalities of cancer (surgery or cytotoxic drugs) in the present study. Moreover, both AF and cancer have been linked with inflammation, and pro-inflammatory factors could be involved [22][23]. In our study hsCRP reflecting inflammation was not increased in either AF or cancer subjects.

It remains unclear, how exactly cancer and AF relates to each other. Due to the original study design of the OPERA study, we lack many specific data on cancer subjects, like the type of cancer and given treatment. Although this study does not shed new light on the understanding of the pathomechanisms of these two conditions, it confirms the relation between AF and cancer. Interesting result was that IMT (intima media thickness) and left atrial diameter were both increased among cancer subjects in our study. Few studies have suggested that anticancer treatment during childhood affects the IMT [24], and in another study IMT was increased in patients with distant metastases [25].

In conclusion, the main finding of our long-term follow-up study was that cancer is an independent risk factor of atrial fibrillation. Additional studies are needed to demonstrate the pathophysiological mechanisms underlying the association between AF and cancer. Understanding the mechanism could help to prevent and treat atrial fibrillation among cancer patients.
